# “Boy, what are we all doing? We are crazy, really crazy”: a qualitative study of psychosocial processes around an atypical one-time smoking cessation course

**DOI:** 10.1186/s40359-023-01448-0

**Published:** 2023-11-20

**Authors:** E. G. Meeuwsen, M. J. J. de Kleijn, E. M. de Boer, A. F. Koçak, N. H. Chavannes, E. Meijer

**Affiliations:** 1 Department of Public Health and Primary Care, Leiden University Medical Center, Hippocratespad 21, Postzone V0-P, PO Box 9600, Leiden, RC 2300 The Netherlands; 2 Independent ‘Health for all’ consultancy PinK-FOX, Tienhoven, The Netherlands; 3 National eHealth Living Lab, Leiden, The Netherlands

**Keywords:** Smoking cessation, Identity, Social support, Long-term Smoking, Group course

## Abstract

**Background:**

Smoking prevalence is still high, which requires effective interventions that help many people who smoke at once in addition to time-consuming individual interventions. ‘I Quit’ is a large-scale smoking cessation course in The Netherlands. This qualitative study explored I Quit participants’ experiences during and after the course, and perceptions of whether and how the course may have altered their smoking behavior.

**Methods:**

We performed individual semi-structured interviews with course participants (*N* = 21) who had either quit successfully, attempted to quit but relapsed, or had continued to smoke after ‘I Quit’. Shortly after qualitative data collection was completed, Foundation I Quit was accused in the media of a number of misbehaviors. Although unplanned, this provided a unique opportunity to explore participants’ views on alleged fraud in a second round of interviews (*N* = 16). Data were collected from 2016 to 2018.

**Results:**

Qualitative findings showed two psychosocial processes that may explain smoking cessation after course attendance. First, the confrontation with a large group of people who smoke, of whom some had already developed smoking-related complaints, triggered identity processes both towards and away from quitting smoking. Unorthodox methods used in the course appeared to trigger identity processes. Second, social support after the course from participants’ own social network facilitated maintenance of successful quitting. The study also found that interview participants’ opinions on I Quit did not change much after allegations of fraud in the media.

**Conclusions:**

Findings suggest that a one-time course might initiate psychosocial processes that could help certain smokers to gain motivation to quit, requiring a minimum of resources. Identity processes triggered by the course seem tricky as people have different ways of dealing with identity threat, some of which can be counterproductive and even result in more difficulty quitting. More research is needed to examine who can benefit from a one-time course, and who needs more support in order to quit successfully.

**Supplementary Information:**

The online version contains supplementary material available at 10.1186/s40359-023-01448-0.

## Introduction

Smoking is the leading preventable cause of death worldwide, resulting in avoidable morbidity and over eight million deaths each year [1]. The harmful effects of smoking are well known, also amongst people who smoke [2]. The vast majority reports regretting smoking and most people who smoke are motivated to quit [2, 3]. Approximately half of those who smoke report having attempted to quit in the past year [3]. There appears to be a trend toward attempting to quit with assistance or supporting interventions, but the majority of quit attempts in populations studies is still unassisted [3–5].

The fact that most quit attempts are unassisted is an opportunity for improvement because the chance of quitting successfully is significantly higher with assistance compared to quitting without assistance. Less than 5% of the unassisted quit attempts result in long-term abstinence [6] whereas people who receive either pharmacological and/or behavioral support are more likely to both successfully quit and to remain abstinent than those who quit without any form of assistance [7, 8]. The chance of long-term abstinence increases by 50 to 130% with group therapy compared to self-help programs, with average quit rates ranging from 9 to 20% [9]. Group therapy is considered as effective as individual counselling and like individual therapy it offers a context for imparting information, teaching skills to cope with cravings and avoid relapse, and to maintain motivation to quit [9]. It also provides opportunity for social support and learning [9].

Currently, most group programs last six to eight sessions [9], but briefer courses exist as well. A quasi-experimental study on a brief group intervention reported a 31.5% abstinence rate after thirteen months, versus 8.3% in the control group [10]. This intervention was provided to small groups by a trained ex-smoker, and mainly aimed to convince smokers that smoking has no real benefits. Another one-time, but large-scale course called “I Quit” was developed by ‘Stichting Ik Stop Ermee’ (‘Foundation I Quit’ in English). I Quit consisted of a four-hour meeting typically held at a large location, such that up to five hundred people could attend at the same time. This course aimed to reduce perceived benefits of smoking by educating the attendees about the tactics of the tobacco industry and the process of quitting, and by providing them with practical instructions for quitting. Taking the minimal trainer-participant contact of four hours and the large number of participants that can attend at once into account, this type of group intervention is promising and could meet the need of the increasing number of people that want to quit smoking.

The aim of this study was to explore experiences of I Quit participants during and after the course, and their perceptions of whether and how the course may have altered their smoking behavior. We conducted individual semi-structured interviews with participants who had quit successfully after the course, as well as participants who had attempted to quit but relapsed, or had continued smoking. I Quit was accused of misbehaviors shortly after qualitative data collection was completed, which provided an unplanned but unique opportunity to explore participants’ views on alleged fraud in a second round of interviews.

## Methods

### Design

Observational qualitative study conducted in the Netherlands. Qualitative data was collected from 2016 to 2018 through individual semi-structured interviews with course attendees. In the first round of interviews (December 2016 – January 2018, approx. 12 months after course participation), we explored participants experiences during and after the course. In the beginning of 2018, Foundation I Quit was accused in the media of a number of misbehaviors. This included fabricating a research report that was supposed to be published by a Dutch university, stating that 81% of course participants successfully quit smoking [11]. In the second interview round (May 2018), we explored participants’ responses to negative media coverage of I Quit. The COREQ checklist was used to report this study.

### Participants and recruitment

I Quit courses were typically organized after a request by a local healthcare centre. Interview participants were recruited via questionnaires that were distributed by local healthcare centres to evaluate abstinence rates among I Quit participants (see Supplementary Materials 1 for Procedures regarding the questionnaires). All course attendees were invited to complete the questionnaires, no further in- or exclusion criteria were specified. Questionnaires were completed in 2016–2017. Interview participants were recruited from the four most recent courses, which were attended by a total of 810 people, 225 of whom gave informed consent to participate in the study, had completed both the initial and follow-up questionnaire and were invited by the study team to participate in an interview. Unsuccessful quitters were purposively recruited to counter selection bias, by sending an additional invitation. Thirty-seven people gave permission for an interview, five people actively refused permission, the other 183 course attendees did not respond. Two of the five people who refused motivated their decision saying they ‘had better things to do’ or ‘not remembering having given permission in the first place’. One participant was excluded because of deafness. None of the researchers had any prior relationships with the participants prior to study commencement.

In total 21 course attendees were included in interview round 1: nine ‘Successful Quitters’ (SQ), all eight ‘Temporary Quitters’ (TQ) and all four ‘Unsuccessful Quitters’ (UQ) that were interested. The remaining fifteen successful quitters were randomly excluded when data saturation was reached. Successful quitters were defined as people who quitted smoking after attending the course and had not started smoking again at the time of the interview. The temporary quitters had tried to quit at least once after attending the course, but started smoking again before the time the interview was conducted. Unsuccessful quitters had not attempted to quit at all since attending the course. Nine participants were male and twelve were female, they were 51 years old on average (range 29–72) and all educational levels were represented. Most participants started smoking around the age of fourteen or fifteen years, and most had been smoking for over twenty years. They all smoked at least ten cigarettes a day before attending the course, four participants smoked between ten to nineteen cigarettes, five smoked between 20–29 cigarettes and ten smoked more than 29 cigarettes a day. Interview participants were rewarded with a twenty-euro gift card after completing the first interview.

All interview participants from round 1 were approached and sixteen of them were interviewed again. Ten of them were female, and they were 53 years old on average. All nine successful quitters, five of the eight temporary quitters, and two of the four unsuccessful quitters participated.

### Procedures and materials

The interviews were conducted by LM by telephone. Interviews and verbal informed consent were recorded separately with a voice recorder. The interviewer introduced herself prior to the interviews with her name and function, and explained that the aim of the interview was to talk about the experiences of the participant with ‘I Quit’. No characteristics of the interviewer were reported to the participants. Interviews were based on a semi-structured interview guide (see Supplementary materials 2). Interviews started with an assessment of the participants’ smoking history and smoking characteristics. Subsequently, participants were asked about their smoking behaviour before and after the course, pros and cons of the course itself, their perspective on smoking and quitting smoking, the group of attendees and about social support or social pressure. Based on existing literature and our own expertise, the interviews also contained some questions about identity [12, 13]. The interviews lasted between twenty-five and forty-five minutes. Interviews in the second round served to explore participants’ views on the negative media coverage of I Quit.

### Intervention

I Quit consisted of a four-hour meeting at which up to five hundred people could attend at the same time. At the time of the study approximately 20,000 people attended every year according to I Quit. The courses were organised in different cities throughout the Netherlands and took place at large locations like churches, conference centres, libraries and cultural meeting places. Attendees paid fifty to one hundred euros for attending the course. In contrast to most group interventions [9], I Quit did not intend to educate attendees about reasons to quit or about the health risks of smoking. Furthermore, at the time when data were collected, the organisation stated that it was not necessary to be motivated to quit before attending. Instead, the course aimed to reduce perceived benefits of smoking by educating the attendees about the tactics of the tobacco industry, the process of quitting and by providing attendees with everyday instructions. The information presented was largely based on documents from the tobacco industry itself, provided by the organisation ‘The Truth’ via www.thetruth.com. The speaker presented the content within a timeframe of only four hours. Most notable is that attendees were encouraged to smoke their last cigarettes during the three smoking breaks, with two cigarettes being smoked right after each other during the last break in order to taste the ‘true taste’ after the need for nicotine was already satisfied. Participants were instructed to quit directly as a group after attending the course. The aftercare provided by the organisation of the course consisted of an e-mail with the course’s content, sent directly after the course had finished and every four months afterwards, providing attendees with tips and tricks to keep them motivated to quit. Attendees who felt at risk of relapsing could use telephonic coaching offered by the organisation. They could call (free of charge) from Monday till Saturday during working hours if they felt tempted to start smoking again, or ask questions by e-mail.

At the beginning of 2018, Foundation I Quit claimed that around 20,000 people attended each year, and that 81% of the participants was still abstinent one year after attending the course [11], but a reliable basis for these numbers was lacking. Based on questionnaire data collected before the current qualitative study, abstinence at about one year follow-up was estimated to lie between 4.4% and 45.3% - with 4.4% likely being pessimistic, and 45.3% likely being overly optimistic (see Supplementary materials 1).

### Analysis

Qualitative analysis followed the Framework Approach [14–17], which combines inductive and deductive analysis. The interviews were conducted by LM, transcribed verbatim by LM (first round) and FK (second round), and coded by LM, who was familiar with the content and context of the answers. Both LM and EM independently created a coding tree, and coding trees were compared for consistency. Intercoder agreement was high but not enough subcategories were differentiated. A final, more detailed coding tree was developed which resulted in seven themes and ninety-two subcategories. Three of the coded transcripts were reviewed by EM, who was experienced in qualitative research. Once all transcripts were coded, themes and categories were refined applying the analytical framework method. Codes were grouped using the program Atlas.ti. Data clusters (families) were interpreted by looking for patterns and identifying answers to the specific research questions performing Cross Case analysis. Interpretations of the data were constantly checked with the data, and discussed between LM and EM in order to ensure reliability of findings. Emerging findings on identity processes resulting from inductive analysis were interpreted using social identity theory [13], PRIME theory [12] and theorizing on possible identities [18] as theoretical frameworks.

Reflexivity was practiced by being aware of, and discussing, how the author’s backgrounds and expertise may have affected data collection and interpretation. The different backgrounds of LM and EM added to the quality of the analysis. LM is a medical doctor, general practitioner in training, and has conducted research into the implementation of a referral strategy for smoking cessation care in general practice. EM is a psychologist, and assistant professor in a university medical centre with a research focus on smoking cessation, lower socio-economic position, identity, behaviour change, and evidence-based smoking cessation methods. She has developed several eHealth interventions for smoking cessation, and was project leader of the Dutch Tobacco dependence clinical guideline. She also works as a psychologist in mental healthcare.

### Patient and public involvement

There was no involvement of patients, public or the foundation I Quit in the design, conduct, or reporting of this study. Moreover, this study was not commissioned by the foundation I Quit but was initiated by the authors. I Quit provided the authors access to their courses to understand the program and to ask the participants to participate in this study.

## Results

### Course entry, expectations, and general evaluation (interview round 1)

***Course entry and expectations.*** Sixteen participants, only successful and temporary quitters, were recommended to attend I Quit by friends, colleges or family members. Four participants read about the course in local media, and seven participants (also) learned about the course by visiting a healthcare professional. Although sixteen participants said they knew the advertised high quit rate, most participants did not have specific expectations. Nine of the sixteen people were skeptical about the advertised quit rate, however this did not prevent them from participating. All successful quitters stated they ‘did not really have any expectations’. Six of the participants, temporary and unsuccessful quitters, on the other hand felt motivated to attend because of the advertised high quit rates. They said they were ‘curious about the course’s secret’, expected ‘being brainwashed’ or were hoping for a ‘miracle’.

***General experiences of I Quit.*** Most participants were, overall, satisfied with I Quit, stating that it was for example ‘perfect’, ‘ideal’, ‘convincing’, and that it was a ‘great evening’. As can be expected, the unsuccessful quitters were less positive. We will present the key points of participants’ general experiences of I Quit below.

With regard to the course’s content, participants were most impressed with learning about the tobacco industry’s tactics, and the ingredients a cigarette contains. Ten participants stated they heard new information about the tobacco industry. They learned how ‘clever’ and ‘sly’ the tobacco industry works, by intentionally making and keeping them addicted. It made many participants feel like they were not ‘weak’, but ‘just addicted’. Being asked what really made the difference in the process of quitting smoking, P18 said:

‘*I think, for me personally, it was the realisation that the marketing of the cigarette, or what’s in a cigarette, what made me think ‘no, I am almost stupid if I let myself being talked into this’. … I just started to make fun of myself, it made me feel stupid. It’s just confrontational. Purely based on a couple of stupid facts…. Because it’s not about how bad smoking is for you and that it can kill you by causing lung cancer, everybody knows that.*’ [P18 TQ].

Participants were also impressed by learning about the history, development and ingredients of the modern-day cigarette, including learning that cigarettes contain substances like ammonia and polonium, and their effects on the body and mind. Many participants stated this was information a smoker simply would not know, without attending the I Quit course.

Participants also mentioned knowing ‘what to expect’ after quitting as an important factor in their success of quitting smoking, as this knowledge seemed to facilitate self-efficacy and motivation, and reduce fear of quitting. Several participants expressed that the chronological presentation of expected symptoms after quitting, accompanied by practical advice, led to ‘recognition’ during the first couple of days after the course took place. Sudden cravings for a cigarette were symbolised as the ‘small or big monsters’. Many participants mentioned these monsters spontaneously during the interviews and used the coping mechanism they were thought to counter these cravings: they would seek distraction for a couple of minutes, knowing the craving would fade. Two successful and two temporary quitters appreciated that the course’s content was based on ‘scientific research’, because if it would not have been based on science ‘anybody could say anything’ [P2]. They said the story made sense and was ‘logical’. P18 stated he felt confident because the speaker referred to studies done by the tobacco industry, as well as studies by doctors. Importantly, the speaker gave the controversial advice not to use any kind of supportive medication when quitting smoking. Seven participants mentioned that this was new to them, but that they were convinced by the speaker’s arguments or found that it matched their own experiences. Several participants stated they ‘learned why their previous quit attempts had failed’ or that ‘all these so called ‘supportive tools actually maintain your addiction’. [P21 TQ]. For example, P9 SQ explained:

*And of course there is this nicotine, this chewing gum I tried once, but that’s not going to work either. “I Quit” made me understand there is plain nicotine in there, so that doesn’t work at all. So yes, slowly you start to understand ‘ah, that’s how it works’.*

However, several participants disagreed with the speaker, such as P14 TQ who stated that ‘*the course gives you the illusion that you can do it on your own… I am sure there are people who are actually able to do so, but I was just far too addicted to that stuff [cigarettes].*’

Participants stated that the smoking breaks helped to lower the threshold to attend, as they would not feel comfortable or would even refrain from participation if they would have to sit still for four hours without smoking. Sixteen participants were clearly enthusiastic about the breaks, which they found for example ‘brilliant’, ‘amazing’, and ‘the course’s strongest point’. However, four of the successful quitters and one unsuccessful quitter thought it strange that a smoking cessation course included smoking breaks. Only one participant had called the telephone number after the course, and most participants did not read the e-mails from the organization.

### Psychological processes related to course attendance (interview round 1)

The analysis resulted in two main themes concerning the psychosocial processes occurring during the course: *social support* and *identity processes*, which will be discussed below. We will also discuss the role of the *smoking breaks* in shaping identity processes.

***Social support.*** Being in one place with so many other people who smoke strongly impressed participants. Initially it reassured some of them, by making them feel like they were ‘not alone’ because ‘you never see so many smokers at one place anymore’ [P16 TQ]. Or, as a successful quitter said, ‘In the end we all attended as fellow sufferers’ [P8 SQ] .

Two of the participants were surprised to see so many other people who were, apparently, motivated to quit smoking. One of them stated that it was ‘alarming’ to see so many other attendees: ‘I had no idea at all, I didn’t know that so many smokers are trying to quit [smoking].’ Someone also mentioned feeling ‘more anonymous’ because of the large crowd, which made it easier to start a conversation with other attendees. Some participants even said that chatting with the other participants was ‘cozy’. According to one of the temporary quitters, attending the course as a group helped to build motivation to quit: ‘When you decide [to quit] on your own, all alone at home, it is not the same as when you are in a hall full of people who all have the same plan.’ [P1 TQ] One other temporary quitter confirmed this by stating that quitting smoking is ‘a heavy burden to carry alone’ [P14 TQ].

However, when asked directly, none of the participants confirmed feeling supported by the large group of attendees, nor did they sense any kind of group pressure to quit. Multiple participants stated that they attended ‘for their own benefit’ and in general, there was no strong sense of belonging to the group. P21 TQ stated that the group of attendees was very diverse, which stood in the way of him feeling connected with the other attendees. One participant even said that the other attendees were skeptical and did not support each other [P18 TQ].

Notably, the large size of the group of other attendees had a clearly negative effect on two of the four unsuccessful quitters. They stated they were used to getting more professional support and to get the opportunity to ask questions during previous supported quit attempts. Attending this large-scale course felt like ‘just business’. For example, P17 (UQ) explained: ‘*I did not feel at ease. I mean I expected it to be a small group. (…) It was terrible [to attend in a large group]. I almost immediately turned around. I thought: this is just mass production. Quitting smoking is such an emotional process for me. A bit smaller and more compact [group] would be good for me.’*

Almost all participants attended the course together with a family member or with friends. Although no one reported feeling supported by the large group of attendees during the course, they often did feel supported by their partners or friends in the period after the course. Many of the successful and temporary quitters felt supported to quit by their close family or friends after the course. Three of them also mentioned ‘not being exposed to cigarettes in the period right after the course made it easier’ not to start smoking again. This contrasts with the unsuccessful quitters, of whom three out of four were still surrounded by people who smoke, like one of them said:

‘*I have no luck, because all of my friends, well they all smoke. When I go to the neighbors’ across the street… they smoke. When I do jobs around someone’s house: the carpenter smokes, the plasterer smokes, the owner smokes. They say ‘fewer and fewer people smoke’, but not in my social circle.*’ [P15 UQ].

In sum, attending the course in a large group made some participants feel reassured as they were ‘not alone’ in quitting, whereas some unsuccessful quitters seemed to feel lost in the crowd. Importantly, no one reported feeling supported or pressured by the other, unfamiliar attendees during the course. However, almost all participants attended together with close friends or family, which was not as important during the course as it was in the period after attending. The successful quitters in particular mentioned experiencing positive support in their ‘social circle’, whereas the unsuccessful quitters mentioned still being exposed to other people’s smoking behavior, which bothered them.

***Identity processes.*** Attending in large groups of ‘fellow sufferers’ also seemed to lead to ‘recognition’, which appeared to help participants to understand their own smoking behavior. This process occurred especially in the group of successful quitters. In total 7/9 of the successful quitters and only 1/8 of the temporary quitters and 1/4 of the unsuccessful quitters identified themselves with the other attendees. Social identification with other attendees was often experienced as disturbing: 5/9 successful quitters described the experience of seeing the other attendees in the hall as ‘confrontational’, ‘appalling’ or ‘sad’. Two of the eight temporary quitters described similar feelings, and one of the four unsuccessful quitters even called it ‘disgusting’ [P13 UQ]. Participants who identified themselves with the other course attendees often condemned their own smoking behavior as well. For example, a successful quitter stated that:

*‘It’s just ridiculous, you absolutely get the idea that we are such strange people. From the very moment we realize we are about to get a smoking break, we can’t sit still. Like a little child that is promised an ice-cream.’* [P9 SQ].

One of the ‘appalling’ factors for multiple participants was their observation that some people who had smoked for a long time could not stop coughing. The speaker seemed to make use of this as well. According to P13 UQ, the ‘established smokers’ who coughed a lot were requested by the speaker to leave the hall during a quiet meditation session at the end of the course. When she discussed her experience of attending the course in a large group, she said:

*It’s disgusting, because everybody is coughing and sneezing. At the end, you’ve even got a moment … music is playing inside and there is this moment of meditating and he [the speaker] will request the people who are coughing terribly, to disappear. That’s when you think to yourself: this is so pathetic.*

Several participants distanced themselves from the ‘established smokers’ who involuntarily expressed ‘typical smokers’ behavior’ like coughing, or had ‘typical smokers’ characteristics’ like a bad skin. Three participants (1 SQ, TQ and UQ each) explicitly volunteered that they did not identify with the other attendees, as they considered themselves to be ‘less heavy smokers’. For example, when describing her experience of attending in a large group, P10 SQ explained:

*‘It’s crazy, you see all kinds of people. And you also see people – and that sounds crazy, but I’m going to say it anyway – with whom you do not want to be associated. You don’t want to belong to that. So, hearing all the coughing … it does something with your mind, it makes you think: ‘Oh! Gross! I don’t want to be part of this anymore’. So, it worked for me. It made me think ‘I don’t want to be a smoker anymore’.’ [P10 SQ]*.

Participants who did not identify themselves with the other attendees either said they felt like they did not belong to the group of attendees, or they referred to the other attendees as ‘them’. They used words like ‘bizarre’ and ‘gross’ in order to describe the ‘typical smokers’ behavior’. As such, it seemed that seeing the other attendees was threatening to these participants’ social identity. In order to maintain a positive sense of self, they distanced themselves from the others both by thinking of ways in which they were different (e.g. ‘less heavy smokers’), and by attempting to quit.

In addition to feelings of threat based on being part of the group of ‘attending smokers’, several participants projected their observations of ‘established smokers’ onto their future selves. For instance, one successful quitter [P5] described that ‘I heard many people with these smokers’ coughs, I thought: ‘Oh god, this is what I will become, you know’… I did not cough like that, but that’s your foreland, that’s where you are heading to.’ When asked how it made her feel to be in this crowd, she said: ‘It works really well! You should add a few of these really old, hard-core smokers to every group. …. I automatically project that onto myself.’ As such, by observing other attendees, this successful quitter had realized that she was heading towards becoming ‘like the established smokers’. This future self, which was clearly undesirable to her, seemed to have helped to build motivation to quit smoking. Similarly, P18 TQ explained that seeing all the other attendees felt ‘very confrontational’ and she felt like she was not part of the group of ‘insecure, older smokers’. She stated that ‘And then I realized: I never want to be like that, never.’ Like P5 SQ, seeing the older attendees seemed to make her think about her future self. When asked whether she fitted in the group, she said: ‘no, absolutely not’. Several interview participants, especially successful quitters, seemed to deal with the threatening ‘established smoker’ future self by making efforts not to end up like the established smokers they saw in the course.

These perceptions that participants had of course attendees, and as such of themselves, resembled experiences of societal stigma forwarded spontaneously by eight participants. In essence, they all described the same change of societal perspective on smoking. Smoking used to be ‘cool’ but they stated that since recent years it was considered to be ‘pathetic’, ‘stupid’, ‘bad’, ‘unhealthy’, etcetera. Three participants felt that people who smoke are currently seen as ‘pariahs’. Most successful and temporary quitters that expressed their opinion about this topic were in favor of the changing social perspective towards smoking, because they perceived it as a beneficial development from a health perspective. Several interview participants, regardless of their success in quitting, had felt social pressure to quit smoking, which was generally experienced as unpleasant. Some participants stated that it helped to quit smoking, but others found it had gone too far, describing the social pressure as a ‘witch hunt’. This seemed to have adverse consequences, for example P21 SQ stated that in the past he was an ‘angry smoker’ who would ‘continue to smoke for the rest of my life, of sheer frustration [with the social pressure].’

*Smoking breaks.* Seeing the other attendees in one big place was already confrontational, however this feeling was magnified during the smoking breaks. As such, it is possible that the identity processes described above were strengthened by the setting in which I Quit took place. The smoking breaks turned out to be a logistic challenge, especially at the doors, the stairs or at the balcony, depending on the courses’ locations. The combination of time pressure and the need for a cigarette led to pushing and elbowing, and caused a feeling of agitation among many participants. This led to amusement for some, such as P12 TQ: ‘It’s just hilarious to see everybody walking outside in order to smoke as soon as possible. Ridiculous! So, we made fun of ourselves too.’ One participant [P2 SQ] -who promptly stopped smoking during the course- described the rush to get outside as a ‘spectacle’ that led him to think that ‘boy we are crazy, really crazy’. The setting where the course took place played a crucial role, because there was a revolving door through which only three people could pass at the same time. He also stated that the speaker made fun of the attendees, for being addicted. In the case of this participant, it led to the following realization:

*‘That man [the speaker] said: ‘We will have a break lasting 15–20 minutes and you will have to smoke outside. So, keep in mind, you will have to get through the revolving door, so good luck.’ On the way outwards, I already had my cigarette ready… we were one of the last to leave the hall. As a consequence, you simply get pushed in the back a few times. I said: ‘Easy guys, calm down, calm down’. And as I am looking at that revolving door with all the hassle and pulling, I think: ‘gosh, that man [the speaker] is right’. I sat down with a cup of coffee, I threw my cigarette away and I thought: ‘I am done with it!’ and I never smoked again.’ [P2 SQ]*.

The combination of the large number of attendees and their urge to get outside to smoke made some participants behave impolitely. Taking into account that the speaker predicted this kind of behavior, it made P2 SQ, who smoked heavily at the time, realize that he did not want to smoke anymore. P5 SQ had a similar experience, and stated that the combination of smoking breaks and the large group was the ‘key to success in smoking cessation’:

*‘The combination of sitting in a hall full of smokers, of whom quite a few have developed let’s say ‘severe COPD’ and their behavior: running outside all together in order to smoke! Leaving the entire hall empty! … Wow, I absolutely thought: my god” (…) That’s when you think: ‘[Curse], what do we behave ourselves in a sad way, terrible. We are slaves to the cigarette.’* [P5 SQ].

Similarly, another successful quitter said: ‘All the people around you that smoke and the smoking breaks. Otherwise, I don’t think it will work. (…) All factors combined might have been the formula to success. It was not fun to smoke. (…) Thinking back, it [the setting] might have been a deliberate choice. I now say: leave it that way!’. [P10 SQ]

In conclusion, several group-related factors seemed to facilitate the process of smoking cessation. First of all, many participants mentioned feeling reassured by the other attendees, their ‘fellow sufferers’. They also experienced a sense of recognition, which initially appeared to work counterproductive, for example because participants thought it was cosy chatting during the smoking breaks. However, because participants did not want to be associated with the other attendees and their ‘herd behaviour’, or projected what they saw in ‘established smokers’ onto their own future selves, many were discouraged to continue to smoke. This process seem to be facilitated by the high number of attendees in combination with short smoking breaks. Participants described that this led to a logistic challenge that magnified the addicted ‘smokers’ behaviour’, which seemed to have motivated seven of the eight successful quitters and four of the seven temporary quitters to quit smoking.

### Response to negative media attention (interview round 2)

Most (14/16) participants had heard about the media coverage of the course, of whom half had heard about reported abstinence rates being incorrect, and some had heard about fraud with study reports. Importantly, participants were not very interested in the news, mostly because they had experienced that the course had helped them and/or people around them. For example, P13 TQ stated that ‘I did not get a negative impression of the course. It was a good course. It’s not smart that they present fictive percentages, but I know that it works.’ The large majority of participants -who had all quit either successfully or temporarily- was still positive about the course and would still advise other people who smoke to attend. Several participants who felt supported by the course appeared to empathize with Foundation I Quit. For example, P4 SQ said: ‘It’s a pity, I think people’s hope is taken away (…) If something that works so positively [the course] at least for me, that you put that in a negative light, that’s a shame’. Some participants who were already negative about the course did not follow the news as they had lost interest in the course altogether, such as P14 TQ who said: ‘It hadn’t helped me, I was done with it, so I thought gee, that’s stupid’. Only one participant P15 UQ reported a more negative opinion on the course since hearing the news. Overall, participants’ opinions on the course did not seem to change after negative media coverage, as their views seemed based more on their own experiences than on news reports.

## Discussion

This study explored the experiences of participants with I Quit among people who had been smoking for a long time, as well as their perceptions of whether and how the course had altered their smoking behavior. Qualitative results provide new insight into how social support and identity processes may occur during and after a mass smoking cessation course such as I Quit, including the role of controversial course components. In addition, as I Quit unexpectedly received negative media attention after initial data collection for this study, we were able to explore how course attendees respond to such media coverage.

Qualitative findings from the first interview round suggest several mechanisms that may explain why people undertook a quit attempt, and some of them quit successfully, after attending I Quit. Interview participants mentioned that the education on tobacco industry tactics, ingredients of cigarettes, and what to expect when quitting was helpful. As such, it is possible that perceived benefits of smoking were lowered, in line with the course’s aims. However, based on participants accounts, other mechanisms seemed more important in participants experiences of the course and how the course might have facilitated a quit attempt. First, many successful and temporary quitters were confronted by their social identification with other attendees, resulting in negative perceptions of people who smoke including themselves (e.g. as stupid, crazy or strange) as well as associated negative emotions (e.g. sadness, disgust). This process is well described by social identity theory, which states that an important part of people’s identity (i.e. their perception of ‘who I am’) is based on their membership of social groups or categories, and that this social identity influences behavior [13]. Social identification with other attendees seemed to be stimulated by certain course characteristics, such as the large group of attendees, hassles during smoking breaks, and the fact that attendees who coughed during the meditation part were asked to leave. A number of interviewees even stated that these characteristics of the course were ‘the key to its success’. However, people who smoked heavily seemed to be labeled negatively and positioned -even physically- as an outgroup, which likely contributes to a stigmatized status [19, 20]. None of the interviewees was requested to leave, such that we are uncertain how these people responded, but previous research into stigma suggests that they may hide their smoking status from healthcare professionals, family and friends, become angry or feel victimized, all of which decrease the odds of a successful quit attempt [19]. Although it can be questioned whether the approach is ethical, the confrontation with other attendees triggered identity processes towards quitting smoking. Participants seemed to protect their positive sense of self by distancing themselves from the group of people who smoke, by undertaking a quit attempt. Several participants projected their views of people who smoke heavily onto their own future selves, which is known to be a strong motivation for behavior change [18]. However, other interviewees dealt with identity threat by making clear statements that they did not want to be part of this group, and by making downward comparisons with people who smoke heavily. This might lead people to conclude that, in comparison, they are doing all right and that no action is needed. The tactics to maintain a positive sense of self that were reported by course participants resemble those reported in the literature on identity processes in the context of smoking [21]. Overall, identity processes triggered by the course seem tricky as people have different ways of dealing with identity threat, some of which can be counterproductive and even result in more difficulty quitting.

Second, and in line with previous findings, this study showed the important role of social support for quitting, which may also explain abstinence [22, 23]. Most successful quitters mentioned experiencing positive support in their ‘social circle’, whereas the unsuccessful quitters mentioned still being exposed to other people’s smoking behavior a lot. This did not play a big role during the course, but it certainly was important in maintaining abstinence after attending the course [24]. Although some participants of I Quit felt like they ‘were not alone’ in the process of smoking cessation because of the large group of attendees, there was no evident feeling of group support or group pressure. This might be because the duration of the course, lasting only four hours, or because the setting did not feel intimate or safe enough because of the large numbers of attendees and some attendees deliberately being stigmatized by the speaker.

Third, several unorthodox methods are being used during the course, like stigmatising smoking behaviour, strongly advising participants to smoke during the course, and to smoke two cigarettes directly after each other at the end of the course. These methods, together with other factors, appear to play an important role in triggering behavioural and identity change processes and thus in the potential success of the course. However, even if these methods contribute to change in some participants, stigmatising people and stimulating people to smoke have obvious downsides and should not be used.

Qualitative data collected in the second round of interviews, collected after negative media coverage of I Quit, sheds light on attendees’ responses to allegations of fraud. Surprisingly, their opinions on I Quit did not change much. This contrasts a recent study on donor’s responses to fraud by non-profit organizations, which showed a subsequent loss of trust in donors, especially if media attention was larger and organizations were not transparent in disclosing fraud [25]. The majority of study participants based their opinion on I Quit more on [26]their own experience or the experience of people around them who attended the course, than on the validity of evaluations of effectiveness as reported in the media. The greater value that people place on their own experience over evidence-based methods might explain why a lot of smokers still use non-evidence based smoking cessation interventions and sometimes even prefer these methods [26]. These findings suggest that recruitment strategies for smoking cessation interventions might make use of experience (e.g. advertising personal stories) in addition to information about the intervention’s effects, as effectiveness is not as relevant to some people who smoke as it is to healthcare professionals and researchers. Interestingly, although some participants appreciated that the course’s content was based on scientific research, the news about alleged incorrect success rates and study reports did not seem to affect participants’ opinions on the course. Notably, although part of the content of I Quit is indeed based on research, I Quit also propagates that pharmacotherapy for smoking cessation is produced by the tobacco industry, and that it is ineffective and should not be used – the latter contradicting the literature and clinical guidelines [27]. Likewise, the course discourages people who cannot quit smoking to visit their general practitioner, whereas the general practitioner has a central position in Dutch smoking cessation care and provides evidence-based counseling.

### Limitations

This study has limitations. First, we are not certain whether identity processes emerged only because of I Quit, as participants also brought up societal developments which may have played a role. However, it seems likely that I Quit itself led participants to reconsider who they are and want to be. Second, participants might have given socially desirable answers in the interviews [28]. We tried to facilitate openness through an empathic and non-judgmental attitude, in order to create a safe and confidential environment in which the participants were interviewed. We also clearly stated that the study was independent of the I Quit foundation. Notably, as is typical in qualitative research, the results are not intended to be generalizable to all course attendees. By including people who either quit successfully, temporarily, or unsuccessfully, we believe that we have captured some of the key psychosocial processes taking place around I Quit.

### Implications

The current findings have practical implications. It appears that a one-time group course might initiate psychosocial processes that could help certain smokers to gain motivation to quit, requiring a minimum of resources. As such, there is potential for local healthcare organizations and governments to implement courses with a large group of attendees. Obviously, such courses should refrain from stigmatizing attendees in order to avoid adverse consequences, provide reliable information on effectiveness, and convey information in line with clinical guidelines. It also appears useful to include general psychoeducation, a discussion of motivation to quit, and quitting strategies [9]. More research is needed to examine effectiveness of such formats, including who can benefit from relatively low-intensity support, and who needs more support in order to quit successfully. Possibly, in addition to facilitating motivation to quit smoking, a one-time course could also be used to connect people who want to quit with more intensive local smoking cessation programs. This may help to identify people who are otherwise not reached, and at the same time reduce burden on general practices to motivate people to quit smoking.

Findings also have implications for research into smoking and identity. Although studies consistently show that people who smoke need to be able to see themselves more as non-smokers and less as smokers in order to quit successfully, less is known about how interventions may facilitate such identity change [20–22, 29–32]. Current findings may inspire the development of identity-based interventions for smoking or other addictive behaviours. For example, video or verbal testimonials can be used to help people who smoke visualise their future selves as both continued smoker and successful quitter, and recordings of the behaviours of groups of people who smoke and those who have quit successfully (e.g. in places where smoking is not allowed) may help people who smoke to reconsider their social identification with both of these groups. Importantly, such materials should not be stigmatising, and people who smoke should be provided with guidance on how to quit smoking successfully.

### Electronic supplementary material

Below is the link to the electronic supplementary material.

Supplementary Material 1

Supplementary Material 2

## Data Availability

Pseudonymized data is available from the corresponding author upon reasonable request.
