# Supplementary material for: “Boy, what are we all doing? We are crazy, really crazy”: a qualitative study of psychosocial processes around an atypical one-time smoking cessation course
Source: BMC Psychol. 2023 Nov 20;11:405. doi: 10.1186/s40359-023-01448-0 (PMC10662623; doi:10.1186/s40359-023-01448-0)
Supplement: Supplementary file 1 — Supplementary Material 1 [file 40359_2023_1448_MOESM1_ESM.docx]

**Supplementary Materials 1: Quantitative evaluation of abstinence rates after ‘I Quit’.**

Quantitative data was collected in two cohorts of participants of I Quit, using an initial self-report questionnaire several weeks after attending to assess participant and smoking characteristics, and a follow-up questionnaire approximately twelve (cohort 1) or fifteen (cohort 2) months after attending the course to assess abstinence. Interview participants were recruited from the four most recent courses of cohort 2.

*Participants and recruitment*

Two subsequent cohorts of smokers who attended I Quit were invited to complete a questionnaire. Data collection took place from October 2014 till June 2015 for cohort 1 (five courses delivered in three municipalities in the Netherlands), and from November 2016 till February 2017 for cohort 2 (eight courses delivered in five municipalities in the Netherlands). Most attendees paid fifty to one hundred euros for attending the course, except for participants of one of the cohort 1 courses that was provided for free by the foundation I Quit. All course attendees were invited to participate, no further in- or exclusion criteria were specified. Interested attendees left their contact details using forms provided at the course location, and indicated whether they wanted to be approached by phone or e-mail. Informed consent and questionnaires were sent online or by post, depending on participants’ preference. Informed consent for study participation was obtained from 117 cohort 1 participants and 179 cohort 2 participants.

*Procedures*

Quantitative data was collected through self-report questionnaires. Both questionnaires in cohort 1 and the initial questionnaire in cohort 2 were administered by post or by e-mail, depending on participant’s preference. The Qualtrics program was used for the follow-up questionnaire in cohort 2. Participants who did not complete the follow-up questionnaire were first reminded by e-mail. We called participants who still did not respond, and offered to complete the questionnaire during the phone call. Prolonged abstinence was assessed with one question, i.e. ‘Did you smoke since participating in the course?’ yes/no.

Quantitative data collection followed the guidelines of the Helsinki Declaration of Good Clinical Research Practice. Both the original research protocol (for cohort 1) and its amendments (for cohort 2, and the second round of interviews) were cleared for ethics by the Medical Ethics Committee of Leiden University Medical Centre (P14.327). All participants signed a written (online) informed consent for quantitative data collection.

*Analysis*

Descriptive statistics were used to assess prolonged abstinence, using both a per protocol analysis and an intent-to-treat analysis, using SPSS version 25. In the intent-to-treat analysis, participants who did not response at follow-up were marked as non-abstinent.

*Results*

Most course participants did not provide informed consent for study participation, complicating the reliable assessment of abstinence rates and course evaluation at follow-up. These findings are likely to be biased due to high attrition. According to Foundation I Quit, 440 and 1411 people participated in the courses grouped as cohorts 1 and 2, respectively. Informed consent for study participation was obtained from 117 (26.5%) cohort 1 participants and 179 (12.6%) cohort 2 participants, such that the study samples are likely to be biased (see Table 1 for participant characteristics). Follow-up data was collected from 110 cohort 1 participants at twelve months after the course, and from 98 cohort 2 participants fifteen months after the course. At follow-up, 53 cohort 1 participants and 63 cohort 2 participants reported abstinence. Compared to the study samples (i.e. people providing informed consent) and adopting an intent-to-treat approach, abstinence was 45.3% in cohort 1 and 35.2% in cohort 2, assuming ‘not abstinent’ for drop-outs. However, compared to the total number of course participants, abstinence rates are 12.0% for cohort 1, and 4.4% for cohort 2. As such, abstinence at follow-up is estimated to lie between 4.4% and 45.3%.

**Supplementary Table 1. Participant characteristics and course evaluation in cohort 1 and 2.**

| **Characteristic** | **Category** | **Cohort 1 (*N*=117)** | **Cohort 2 (*N*=179)** |
| --- | --- | --- | --- |
| Age in years, mean (SD) |  | 51.2 (12.1) | 51.84 (12.45) |
| Gender, % (n) | Male | 41.9 (49) | 44.2 (76)^a^ |
| Educational level, % (n) | Lower | 14.5 (17) | 26.3 (47) |
|  | Average | 25.6 (30) | 43.0 (77) |
|  | Higher | 59.8 (70) | 30.7 (55) |
| Age at smoking onset, % (n) | ≤14 | 27.4 (32) | 40.2 (72) |
|  | 15-16 | 41.0 (48) | 35.8 (64) |
|  | ≥17^a^ | 31.6 (37) | 24.0 (43) |
| Years of smoking, % (n) | < 5 | 0.0 (0) | 1.0 (2) |
|  | 5-10 | 1.7 (2) | 2.7 (5) |
|  | 11-20 | 15.4 (18) | 12.3 (23) |
|  | >20 | 82.9 (97) | 79.7 (149) |
| Previous quit attempts, % (n) | Never | 18.8 (22) | 7.8 (14) |
|  | 1-3 times | 54.7 (64 ) | 56.4 (101) |
|  | ≥4 times | 26.5 (31) | 35.8 (64)^b^ |
| Cigarettes per day before attending the course, % (n) | ≤ 9 | 16.2 (19) | 10.6 (19)^c^ |
|  | 10-19 | 37.6 (44) | 39.1 (70) |
|  | ≥20 | 46.2 (54) | 48.6 (87) |
| Course recommendation, % (n) | Certainly | 69.2 (81) | 72.6 (130) |
|  | Probably | 23.1 (27) | 24.6 (44) |
|  | Probably not | 6.0 (7) | 2.8 (5) |
|  | Certainly not | 1.7 (2) | 0 (0) |
| Course grade (1-10)^d^, median (IQR) |  | 9.0 (8.0-9.0) | 8.0 (8.0-9.0) |
| *Note.* Data are means (standard deviation), percentages (number), or valid percentage (number/total number of patients excluding missing data). IQR = interquartile range.  a. Missing for 7 participants.  b. “I am not sure” was recoded into ≥4 times.  c. 2 people did not smoke at the time, and 1 person smoked cigars.  d. 10 indicates best possible grade. | | | |
