# Supplementary material for: “Boy, what are we all doing? We are crazy, really crazy”: a qualitative study of psychosocial processes around an atypical one-time smoking cessation course
Source: BMC Psychol. 2023 Nov 20;11:405. doi: 10.1186/s40359-023-01448-0 (PMC10662623; doi:10.1186/s40359-023-01448-0)
Supplement: Supplementary file 2 — Supplementary Material 2 [file 40359_2023_1448_MOESM2_ESM.docx]

**Supplementary Materials 2: Interview protocols round 1 and 2.**

# Interview protocol round one

1. Introduction, explanation, informed consent
2. Smoking history
   1. At what age did you start smoking? (why did you start smoking?)
   2. How much did you smoke?
   3. Have you ever tried to quit smoking before participating in the course “I Quit”?

(Ask about all attempts to quit or reduce smoking)

If so:

- - 1. How often and for how long?
    2. Did you use (other) methods of cessation support (like for example a website, book, app, medication, meditation, etc.?)
  1. Why did you want to quit smoking?

1. Smoking behaviour since participating in the course
   1. Did you quit smoking after attending the course?
   2. If yes:
      1. When did you quit?
      2. Are you still nonsmoking?
   3. If participant still smokes:
      1. When did you start smoking again? (ask about all quit attempts if applicable)
      2. Did you decrease your smoking?
2. Before the course
   1. How did you hear about the course? (Did you orientate yourself in advance? For example, by looking on the internet/social media, if so: what did you read about the course?)
   2. When you started the course, did you know the success rates/chances of quitting smoking? If so, what did you think of that?
   3. Why did you participate in the course? / What made you decide to participate in the course?
   4. How did you feel about quitting smoking before participating in the course?
3. Course “I Quit”
   1. What were your expectations of the course beforehand?
   2. What did you think of the course afterwards?
   3. What did you think of the course design? (e.g. smoking break, presentation)
   4. Did you get new insights? If so:
      1. What was a real ‘eye-opener’ for you?
   5. Which aspects of the course did you like?
   6. Which aspects of the course could be improved in your opinion? What would an ideal course look like?
   7. How did you feel about participating in a group?
      1. How big was the group of participants?
      2. Was the group size important to you?/Did the size of the group matter to you?
      3. Did you already know other course participants beforehand? If so, what was your relationship to these people?
      4. Did you experience support from other participants? If so, in what way? If not, why not?
      5. If applicable: did you feel free to tell your story in the group?
      6. Did you experience peer pressure to quit smoking/or continue smoking? If so, in what way? If not, why not?
      7. Did you have contact with other participants after the course? If so, in what way? Did they play a role in quitting smoking/resuming smoking?
   8. How did you feel about quitting smoking at the end of the course?
   9. Did you use the helpline the course offered? If so, can you describe when?

In case the participant has **quit successfully**

1. How come you have quit smoking?
   1. Did the course help you to quit smoking? If so, how?
   2. Would you have quit successfully without the course?
   3. Did the group play a role in (quitting) smoking? If so, how?
   4. If the participant knew other participants:
      1. Did they play a role in (quitting) smoking after the course? If so, how?
   5. Have you used other aids to quit smoking? If yes which one?
   6. If you have quit smoking before: was this quit attempt different from previous quit attempts? If so, in what way?
2. What is it like for you to have quit smoking?
   1. What is going well?
   2. Is there anything you find difficult?
   3. What do you think about smoking now?
      1. Do you think smoking fits with who you are?
      2. Do you think non-smoking fits with who you are?
      3. Has this changed?
   4. What is the biggest change for you now that you quitted smoking?
   5. How do you see the future concerning smoking?

In case the participant has continued **smoking or relapsed**

1. Did the course motivate you to quit smoking? If so, how?
   1. What made you continue to smoke or start smoking again?
   2. Did the group play a role in smoking cessation or continuing smoking? If yes, how?
   3. If the participant knew other participants:
      1. Did they play a role in (quitting) smoking after the course? If so, how?
   4. **If the participant attempted to quit**: Have you used other aids to quit smoking? If yes which one?
   5. **If a quit attempt was made had stopped before:** Was this quit attempt different from previous quit attempts? If so, in what way?
2. What is it like for you to smoke again, or to still smoke?
   1. How do you feel about smoking (again or still)?
   2. What do you think about smoking now?
      1. Do you think smoking fits with who you are?
      2. Do you think non-smoking fits with who you are?
      3. Has this changed?
      4. Do you think smoking suits who you are? If so, why?
   3. How do you see the future in relation to smoking?
      1. Would you want to quit smoking?
      2. What do you think it takes to successfully quit smoking?
3. Closing
   1. Do you have any additional comments or questions? Or are there important factors for you that I have not asked you about?

# Interview protocol round two (after media coverage)

*Preparation*Identify participants who quitted smoking successfully (group 1); who quitted temporarily (group 2); who did not quit smoking (group 3)

1. Introduction, explanation, informed consent
2. Media coverage and attitude course
   1. Did you follow the media coverage of the course “I Quit”? *(Note: if participant has questions about this, explain at the end of the interview)*

**If yes (skip if no):**

- - 1. What have you heard about the course “I Quit”?
    2. What did you think of this media coverage?
    3. Have you talked about these news items?
       1. With whom?
       2. What did you discuss?
       3. Did this (discussion about media coverage) affect your opinion on the course?
  1. How do you feel about the course now?

1. Smoking status
   1. **Group 1:**

Have you still quit smoking?

- - 1. If yes: How are you doing having quit smoking?
    2. If no: When did you start smoking again? What was the reason you resumed smoking?
  1. **Group 2 and 3:**

Do you currently smoke?

- - 1. If yes: Has anything changed in your smoking behavior since the previous interview? If so, what and what was the reason?
    2. If no: When did you quit? What was the reason and how are you doing quitting smoking?

1. Course’s role in quitting smoking

*If the participant is still abstinent:*

- 1. Did the course help you to quit smoking? And if so: how?

*If the participant smokes:*

- 1. Did the course motivate you to quit smoking? And if so: how?

1. Attitude smoking/quitting smoking
   1. What do you think about smoking?
   2. What do you think about quitting smoking?
2. Identity
   1. Do you think smoking fits with who you are? Why?
   2. Do you think non-smoking fits with who you are? Why?
3. Smoking cessation care
   1. How do you feel about smoking cessation courses and treatments in general?

(if necessary: ask about different types of available smoking cessation care like group courses, individual support from a general practitioner or a coach, nicotine replacement therapy and medication)

- 1. Suppose an acquaintance tells you that he or she wants to quit smoking, what would you advise him/her to do?

1. Closing
   1. Is there anything else you would like to say about this topic?
   2. If applicable, explain media coverage and explain independent role of researchers
